# Results of pollicisation: what works and what doesn’t

**DOI:** 10.1186/1753-6561-9-S3-A12

**Published:** 2015-05-19

**Authors:** Michael Tonkin

**Affiliations:** 1 Department of Hand Surgery, University of Sydney, New South Wales, 2006 Australia

## 

The aim of pollicisation is to provide a strong, stable and mobile thumb of optimal size and shape. The controlling factors are the quality of the index finger, the quality of the adjacent digits and the stability and mobility of the wrist, all of which will be compromised by hypoplasia of bones, joints and soft tissues. However, even a stiff, bent, hypoplastic index finger accompanying a forearm radial longitudinal deficiency is used as a thumb for some activities, albeit in a modified manner.

Assessment modalities include those of strength, pinch and grip, mobility, functional tests such as the Jebsen timed test, and documentation of thumb use for small, medium and large sized objects.

A number of factors relating to technique will assist the surgeon in obtaining optimal results:

## Incisions

These take the form of a modified z-plasty. The palmar transverse incision is altered in position according to the degree of stiffness of the joints of the index finger. The incisions must allow adequate dissection of the neurovascular bundle of the web space between index and middle fingers and allow adequate mobilisation of the tendons to the PIP joint level.

## Palmar dissection

The neurovascular bundle is identified, directing attention to the possibility of a neural ring or aberrant vascular anatomy. The A1 and A2 pulleys are divided. The intrinsic dissection is performed with the extensor tendons intact and the skeleton intact. Avoid injury to the collateral ligaments at MCP joint level during mobilisation of the intrinsic tendon apparatus and free the lateral bands from the central slip to beyond the PIP joint level.

## Dorsal dissection

Veins and nerves are protected. The extrinsic tendon mobilisation is continued. The extensor tendons are divided at the MCP joint level.

## Bone preparation

An osteotomy is performed at the head-neck junction of the metacarpal, removing the physis, flowering the metaphysis to retain some corticocancellous bone, extending the MCP joint and placing an antegrade K-wire axially along the line of the digit. The diaphysis of the metacarpal is then removed, retaining the base of the metacarpal, leaving the base long on its dorso-radial aspect. The digit is then concertinaed into the defect and held with one 0.7mm K-wire and one 2-0 Ticron suture, along with bone graft to aid in union of the new trapezium to the metacarpal base. Positioning of the ray must satisfy the demands of adequate rotation, palmar and radial abduction, extension of the MCP joint of the index finger, placement of the thumb ray in an anterior plane to that of the finger metacarpals and it is this surgeon’s intent to obtain bone to bone union of the new trapezium to the base of the metacarpal.

## Tendon reconstruction

The extensor indicis proprius is transferred to the extensor pollicis brevis and the extensor digitorum communis to the abductor pollicis longus. A concertina of the lateral bands will allow appropriate tension for the intrinsic tendon reconstructions.

Finally the flaps are decompressed proximally to allow insertion for optimal contour.

## Rehabilitation

The hand and forearm are retained in a plaster splint for five weeks when the K-wire is removed. The therapists can then perform some massage and bathing, along with the parents, using an intermittent functional splint. Buddy strapping of the fingers together will assist in encouraging thumb activity.

A recent study has indicated that union of the new trapezium to the base of the metacarpal is desirable, improving strength and stability of the thumb ray, allowing optimal growth and optimal function.

Precise attention to the above details will provide optimal results.

